# HMNPPID—human malignant neoplasm protein–protein interaction database

**DOI:** 10.1186/s40246-019-0223-5

**Published:** 2019-10-22

**Authors:** Qingqing Li, Zhihao Yang, Zhehuan Zhao, Ling Luo, Zhiheng Li, Lei Wang, Yin Zhang, Hongfei Lin, Jian Wang, Yijia Zhang

**Affiliations:** 1 College of Computer Science and Technology, Dalian University of Technology, Dalian, 116024 China; 2 Beijing Institute of Health Administration and Medical Information, Beijing, 100850 China

**Keywords:** Protein–protein interactions, Human malignant neoplasms, Database

## Abstract

**Background:**

Protein–protein interaction (PPI) information extraction from biomedical literature helps unveil the molecular mechanisms of biological processes. Especially, the PPIs associated with human malignant neoplasms can unveil the biology behind these neoplasms. However, such PPI database is not currently available.

**Results:**

In this work, a database of protein–protein interactions associated with 171 kinds of human malignant neoplasms named HMNPPID is constructed. In addition, a visualization program, named VisualPPI, is provided to facilitate the analysis of the PPI network for a specific neoplasm.

**Conclusions:**

HMNPPID can hopefully become an important resource for the research on PPIs of human malignant neoplasms since it provides readily available data for healthcare professionals. Thus, they do not need to dig into a large amount of biomedical literatures any more, which may accelerate the researches on the PPIs of malignant neoplasms.

## Background

The research on protein–protein interactions (PPIs) is critical to understand how proteins function within the cell. Therefore, hundreds of thousands of PPIs generated by high-throughput methods such as yeast two-hybrid screening and affinity purification coupled to mass spectrometry have been collected together in specialized biological databases such as Database of Interacting Proteins (DIP)^1^ [1], Biomolecular Interaction Network Database (BIND)^2^ [2], IntAct^3^ [3], Human Protein Reference Database (HPRD)^4^ [4], and Biological General Repository for Interaction Datasets (BioGRID)^5^ [5]. However, these high-throughput methods are associated with high error rates (both false-positive and false-negative rates). For example, some genome-wide screens might be associated with false-positive rates exceeding 50% [6–9]. On the other hand, the rapidly growing biomedical literature provides a significantly large and readily available source of PPI interaction data and numerous PPIs have been manually curated by biomedical curators into the PPI databases [10, 11].

Furthermore, PPI data is used globally for the prediction of protein properties, systematic network analysis, and evaluation of novel datasets of PPIs produced in a high-throughput fashion [12]. To this goal, several integrated PPI databases have been constructed. For example, HIPPIE^6^ (Human Integrated Protein–Protein Interaction rEference) is a human PPI dataset with a normalized scoring scheme that integrates multiple experimental PPI datasets including DIP, IntAct, BIND, HPRD, BioGRID, Molecular INTeraction database (MINT)^7^ [13], and MIPS^8^ [14]. The HIPPIE web tool allows researchers to conduct network analyses focused on likely true PPI sets by generating subnetworks around proteins of interest at a specified confidence level. IID^9^ (Integrated Interaction Database) is an online database of known and predicted eukaryotic protein–protein interactions in 30 tissues of model organisms and humans, which covers six species (*S*. *cerevisiae* (yeast), *C*. *elegans* (worm), *D*. *melanogaster* (fly), *R*. *norvegicus* (rat), *M*. *musculus* (mouse), and *H*. *sapiens* (human)) and up to 30 tissues per species [15]. The STRING^10^ database consolidates known and predicted protein–protein association data for a large number of organisms [16]. Apart from collecting and reassessing available experimental data on protein–protein interactions, and importing known pathways and protein complexes from curated databases, interaction predictions are derived from the following sources: (i) systematic co-expression analysis, (ii) detection of shared selective signals across genomes, (iii) automated text mining of the scientific literature, and (iv) computational transfer of interaction knowledge between organisms based on gene orthology.

In addition, there are also some protein-pathway association databases. For example, PathDIP^11^ integrates data from 20 source pathway databases, “core pathways,” with physical protein–protein interactions to predict biologically relevant protein-pathway associations, referred to as “extended pathways” [17].

Since the dysfunction of some PPIs leads to many diseases (e.g., cancer), the analysis of PPI networks has become one of the powerful approaches to elucidate the molecular mechanisms underlying the complex diseases on the system level [18, 19]. Some efforts have been made to construct the cancer-related PPI databases. Among others, CancerNet^12^ is a cancer-specific database that provides cancer-specific molecular interaction networks across multiple cancer types [20]. Currently, 33 human cancer types are included. The interactions contain PPIs, miRNA-target interactions, and miRNA-miRNA synergistic interactions. Experimentally detected PPIs were assembled from five major PPI databases (BioGRID, DIP, HPRD, IntAct, and MINT) and miRNA-target interactions were considered as the combination of the predicted targets from six algorithms and two experimentally validated data sets. Human Cancer Pathway Protein Interaction Network (HCPIN)^13^ is a collection of proteins from cancer-associated signaling pathways together with their protein–protein interactions [21], which was constructed by combining proteins from seven KEGG (Kyoto Encyclopedia of Genes and Genomes)^14^ [22] classical cancer-associated signaling pathways together with protein–protein interaction data from the HPRD. Reference [23] constructed initial networks of protein–protein interactions involved in the apoptosis of cancerous and normal cells by use of two human yeast two-hybrid data sets [24, 25] and four online interactome databases such as BIND, HPRD, IntAct, and Himap [26]. Their method allows identification of cancer-perturbed protein–protein interactions involved in apoptosis and identification of potential molecular targets for the development of anti-cancer drugs.

Currently, the PPIs in these cancer-related PPI databases are manually extracted and curated by human experts from literatures. However, since the number of biomedical literatures regarding PPIs is growing at an explosive speed, automatically extracting PPIs from the literature is adopted to improve the efficiency of PPI information extraction.

To this end, in this work, a Human Malignant Neoplasm Protein–Protein Interaction Database (HMNPPID) was constructed, whose data was extracted by an automatic PPI extraction tool, named PPIExtractor [27], from a large number of PubMed^15^ abstracts involving human malignant neoplasms. The main contributions of our work are listed as follows. First, HMNPPID provides the readily available PPIs of specific malignant neoplasm for healthcare professionals, which can boost the efficiency of the PPIs research of human malignant neoplasms. Then, HMNPPID can hopefully become an important resource for this research. In addition, we provided a visualization program VisualPPI to help the experts analyze the PPI networks of specific malignant neoplasms and thus discover the molecular mechanisms behind them.

## Implementation

### The protein–protein interaction extraction system for biomedical literature

The number of biomedical literatures involving PPIs is increasing at an explosive speed and, for PPI database curators, it is extremely difficult to curate them efficiently. Therefore, we have developed PPIExtractor in our previous work to automatically extract the PPIs from biomedical literature [27]. Given a MEDLINE abstract, PPIExtractor first applies feature coupling generalization (FCG) [28] to tag protein names in text, next uses the extended semantic similarity-based method to normalize them, then combines feature-based, convolution tree and graph kernels to extract PPIs. To our knowledge, PPIExtractor is the first PPI extraction system publicly available which integrates named-entity recognition (NER), normalization, PPI extraction, and visualization. In addition, the technique used in each stage of PPIExtractor can achieve state-of-the-art performance. Therefore, PPIExtractor was utilized to extract the PPIs of human malignant neoplasm from biomedical texts in this work.

### The extraction of PPIs of malignant neoplasms

According to the International Classification of Diseases (ICD) uniform method established by World Health Organization (WHO) and according to the disease etiology, pathology, clinical presentation, anatomical location, and other characteristics, ICD-10 version 2016 (https://browse10/browse10/2016/en) classifies the diseases, making them an orderly combination and representing them with the coding method. According to the classification in ICD-10, we chose 171 kinds of malignant neoplasms (they are listed on the web site http://202.118.75.18:8082/HMNPPID.asp and divided into 13 categories as shown in Table 1), then downloaded their related PubMed, and finally extracted the PPIs from these abstracts using PPIExtractor.

**Table 1:** Classification of human malignant neoplasms in ICD-10 version: 2016

Column no.	Column name	Remarks
1	PubMed ID	The PubMed abstract ID from which the PPI is extracted
2	Protein Name1	The name of the first protein
3	Protein ID1*	The Entrez Gene id of the first protein
4	Protein Name2	The name of the second protein
5	Protein ID2*	The Entrez Gene id of the second protein
6	Confidence score	The score of the PPI assigned by PPIExtractor which reflects the confidence degree of the PPI
7	Related sentence	The sentence including the PPI

To obtain the relevant abstracts of all these malignant neoplasms, constructing the accurate query string for PubMed search is the first step. For example, the query string for the disease *Malignant neoplasm of lung* is “((Malignant AND neoplasm) OR cancer) AND lung AND protein.” The second step is to retrieve the relevant abstracts from PubMed using the query string. In addition. the filters “Humans” and “English” are activated to obtain only English abstracts associated with human species, and the query time is set as December 1, 2015. In the last step, the downloaded abstracts are input into the PPIExtractor to extract the PPIs. Each PPI is assigned a confidence score by PPIExtractor to reflect its reliability. Usually with a confidence score equal to or greater than zero, one PPI can be regarded as reliable. However, in HMNPPID, the PPIs with the confidence scores higher than − 0.6 are retained since, due to the complexity of natural language expression, PPIs with the confidence scores less than 0 may be true ones. The reason why the threshold is − 0.6 is that, in our previous study of protein complex detection in PPI networks [29], the introduction of the PPIs higher than − 0.6 into the original PPI networks achieved the best results in the experiments. In addition, the interactions between two identical proteins were filtered out.

### File format

In HMNPPID, two PPI file types (i.e., text and Excel formats) are provided for each malignant neoplasm. As shown in Table 2, Each PPI record contains seven columns, including the sentence from which the PPI was extracted with which users can also judge the confidence degree of the PPI according to the sentence by themselves besides the confidence score assigned by PPIExtractor.

**Table 2:** The columns included in the PPI record

Column no.	Column name	Remarks
1	PubMed ID	The PubMed abstract ID from which the PPI is extracted
2	Protein Name1	The name of the first protein
3	Protein ID1*	The Entrez Gene id of the first protein
4	Protein Name2	The name of the second protein
5	Protein ID2*	The Entrez Gene id of the second protein
6	Confidence score	The score of the PPI assigned by PPIExtractor which reflects the confidence degree of the PPI
7	Related sentence	The sentence including the PPI

*The protein names that are not normalized by PPIExtractor to any Entrez Gene id are assigned with the ID 0000

## Results

### Overview of HMNPPID

According to the classification in ICD-10 (version 2016), we extracted the PPIs of 171 kinds of human malignant neoplasms and obtained a total of 266,107 PPIs (with threshold − 0.6). By contrast, the number of PPIs with a confidence score greater than or equal to zero is 72,866. The number of specific neoplasm related abstracts downloaded from PubMed and the number of the PPIs extracted from those abstracts can be found on the web site.

**Fig. 1:**
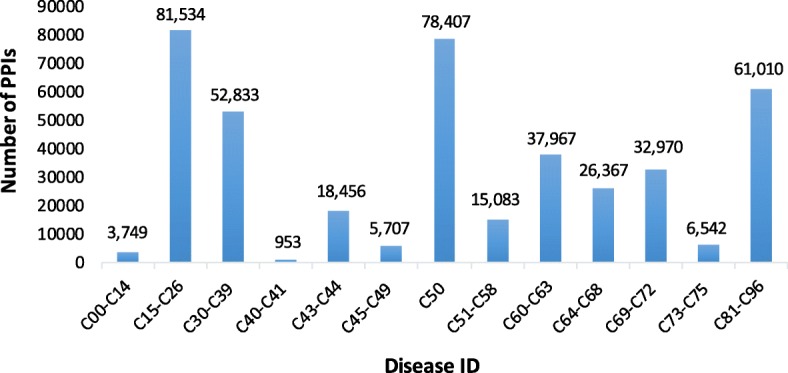
Numbers of the PPIs of 13 categories of human malignant neoplasms

**Fig. 2:**
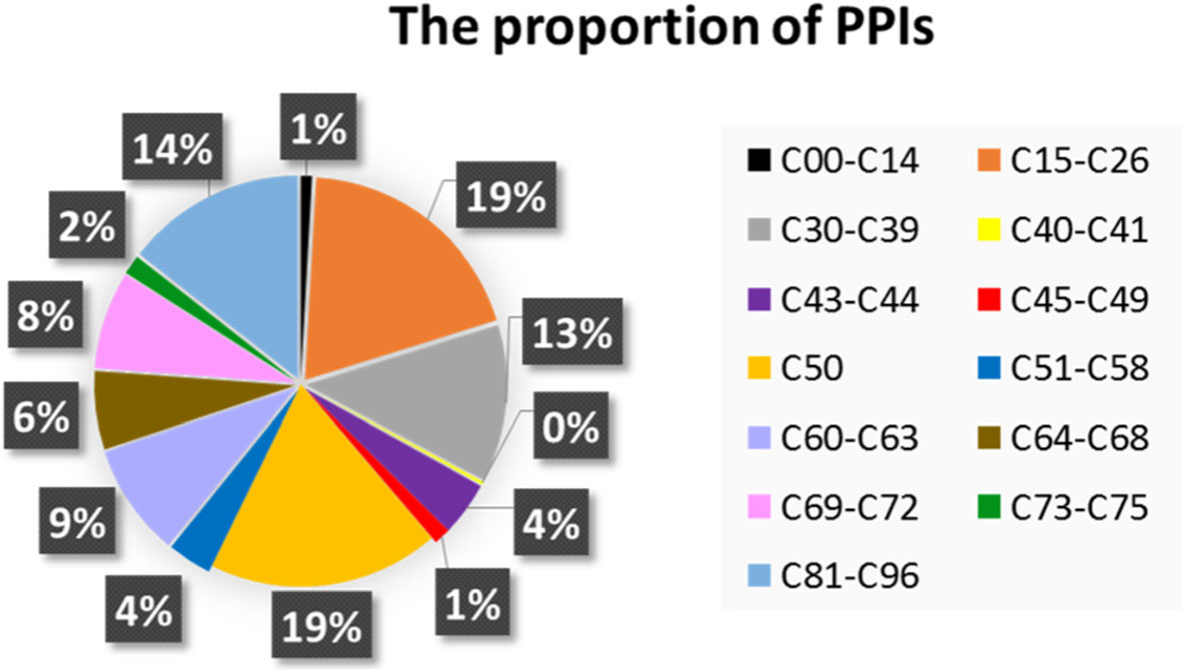
Proportion of the PPIs of 13 categories of human malignant neoplasms. The 13 categories are differentiated by different colors

Figures 1 and 2 show the numbers and proportions of the PPIs of different malignant neoplasms, respectively. As can be seen from the figures, there is a significant difference among these malignant neoplasms. For example, *malignant neoplasms of digestive organs* (C15-C26), *breast* (C50), and *stated or presumed to be primary*, *of lymphoid*, *hematopoietic and related tissue* (C81-C96) have much more PPIs than *malignant neoplasm of bone and articular cartilage* (C40–C41).

**Fig. 3:**
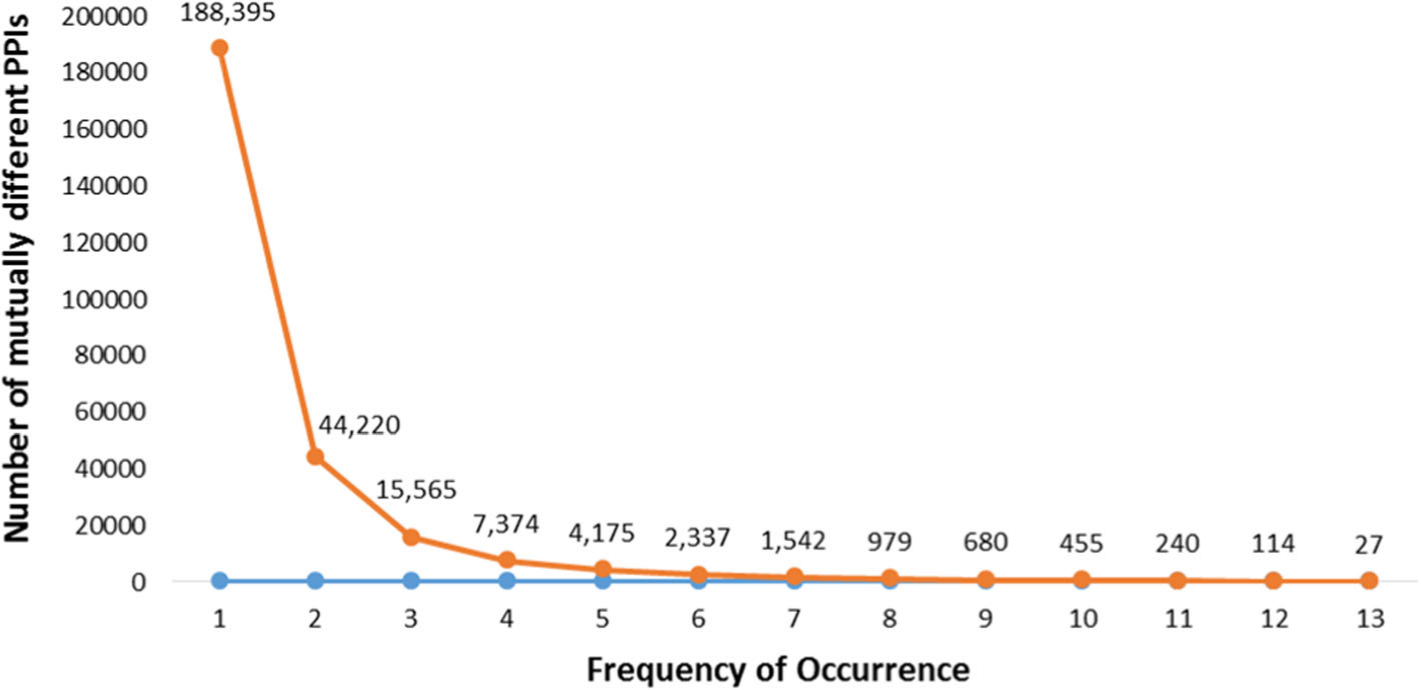
Number of the PPIs with different occurrence frequency. The *x*-axis denotes the occurrence frequency and the *y*-axis denotes the number of unique PPIs

In addition, the occurrence frequencies of unique PPIs in 13 categories of malignant neoplasms are presented in Fig. 3. The majority of PPIs are only associated with a particular category (i.e., the occurrence frequency of the PPI is one). 44,220 PPIs are associated with any two categories; 15,565 PPIs associated with any three categories; 7,374 PPIs associated with any four categories of malignant neoplasms. It is noteworthy that, as shown in Table 3, 27 PPIs are relevant to all 13 categories. Such PPIs tend to be more valuable for healthcare professionals since they may have a biological relation with more malignant neoplasms than others. For example, *p53* has been described as “the guardian of the genome” because of its role in conserving stability by preventing genome mutation [30]. The combination of *p53* and *MIB-1* demonstrates prognostic significance in male germ cell tumors [31] and human bladder tumors [32] (row 2 in Table 3). Activated *p53* binds DNA and activates expression of several genes including *WAF1/CIP1* encoding for *p21* and hundreds of other downstream genes [33] (row 3 in Table 3). Overexpression of *p53* and *Ki-67* could be used to discriminate low-risk luminal A subtype in breast cancer [34] (row 4 in Table 3). *p53*, *cathepsin D*, and *B cell lymphoma 2* (*Bcl-2*) are joint prognostic indicators of breast cancer metastatic spreading [35] (row 5 in Table 3). In addition, *ribosomal S6 kinase 1* (*S6K1*) is a downstream component of the *mammalian target of rapamycin* (*mTOR*) signaling pathway and plays a regulatory role in translation initiation, protein synthesis, and muscle hypertrophy [36] (row 6 in Table 3).

**Table 3:** The PPIs related to all 13 kinds of malignant neoplasms

ProteinName1	ProteinID1	ProteinName2	ProteinID2
p53	7157	MIB1	57534
p53	7157	WAF1/CIP1	1026
p53	7157	Ki-67	4288
p53	7157	bcl-2	596
mTOR	2475	S6K1	6198
Bcl-2	596	Bcl-x	598
Bcl-2	596	Bax	581
telomerase	23293	TERT	7015
CD3+	64231	CD8	925
CD3+	64231	CD4+	920
CD4	920	CD8	925
CD34	947	vimentin	7431
IL 1 alpha	3552	IL 6	3569
IL-1 beta	3553	TNF-alpha	7124
phosphatidylinositol 3-kinase	5295	PI3K	5290
vimentin	7431	cytokeratin	3859
MIB-1	57534	Ki-67 antigen	4288
CTNNB1	1499	WNT	7471
Pgp	5243	MRP1	4363
ERK1/2	5595	JNK1/2	4939
Fas/FasL	355	Fas	356
cytokeratin	3859	AE1/AE3	6521
transforming growth factor-beta	654	TGF-beta	7043
ER	2099	/PR	5541
interferon alpha	3451	IFN	3439
IgG	2217	IgM	959
vimentin	7431	actin	86

### Evaluation of HMNPPID data

For a PPI database, the quality of its data is of great importance. However, there is no cancer-relevant PPI gold set currently. To assess the quality of the data in HMNPPID, we firstly explored the performance of PPIExtractor using the PPIs in HPRD, since the PPIs in HPRD were also collected from the literatures and their reliability is justified (they are curated by expert biologists) and the comparison with it is meaningful. HPRD includes 39,240 PPIs obtained from a set of published articles. We used PPIExtractor to extract 54,808 unique PPIs with the threshold 0 from the abstracts of the same article set (since the full texts of many articles are not available publicly, we only used the abstracts) and 12,870 of HPRD PPIs (accounting for 32.8% of total HPRD PPIs) were matched.

We further analyzed some of the results to find the recall error types. The PPIs in HPRD were curated by expert biologists from both abstracts and full text. Since PPIExtractor was applied only on the abstracts, the PPIs present in the full text were missed out. This accounts for about 68% of total recall errors. In addition, some PPIs in HPRD were extracted by PPIExtractor but with a threshold less than zero (accounts for about 21% of total recall errors). The reason is that due to the complexity of the protein interaction expression, PPIExtractor may fail to extract some true PPIs. In fact, if the threshold is relaxed to − 0.6, almost half (48.08%) of HPRD PPIs could be extracted.

Finally, the names of the proteins of HPRD PPIs are the formal ones assigned by expert biologists which usually are not the same with those used in texts. For example, for a HPRD PPI (***INSR*** 00975 NP_000199.2 ***FABP4*** 02698 NP_001433.1 in vitro; in vivo 1648089), it can be extracted from the sentence “Kinetic analysis indicated that stimulation of ***ALBP*** phosphorylation by *insulin* was attributable to a 5-fold increase in the Vmax…” in the abstract with PubMed ID 164808. ***ALBP*** is an alias of ***FABP4*** (*fatty acid-binding protein 4*) and *insulin* refers to *insulin receptor*, an alias of ***INSR***. However, the failure of matching *insulin* with ***INSR*** by the matching program leads to the recall error of this HPRD PPI. Such errors account for about 11% of total recall errors.

Furthermore, to assess the quality of the data in HMNPPID, we compared it with PPIs in HCPIN. There are 9,784 PPIs among HCPIN proteins. However, since these PPIs are not available, we reconstructed them from the PPIs of seven pathways (i.e., apoptosis, cell-cycle, Janus kinase, mitogen-activated protein kinase, PI3K, transforming growth factor, Toll-like receptor) provided on HCPIN website (http://nesg.org:9090/HCPIN/ShowPathway.jsp) and only a total of 5,815 PPIs were obtained. As a result, 1636 PPIs of HCPIN (accounting for 28.13% of a total of 5815) were found in HMNPPID (72,866 PPIs with confidence scores greater than or equal to zero). Similar to the case of HPRD, the mismatching between the protein names in texts with the ones in HCPIN results in many recall errors.

Considering that the PPIs in HMNPPID were extracted from abstracts rather than full texts, the coverage rates (about 30%) of HMNPPID data with HPRD and HCPIN are still acceptable.

What is more, the 39,240 PPIs in HPRD were curated by expert biologists from 20,074 articles, which means less than two PPIs were curated from one article on average. In fact, only one PPI was curated from one article in most cases. This shows that expert biologists usually only curate the few novel PPIs while ignoring many other PPIs in the article. In contrast, PPIExtractor will extract all the PPIs in the abstracts into HMNPPID, which is especially useful for the researchers who need to explore the relations between the multiple PPIs from one single article or a set of related articles (i.e., these PPIs are usually associated with each other). This is also the reason why PPIExtractor can extract more PPIs than HPRD from the same article set (54,808 vs 39,240). However, the quality of the PPI data in HMNPPID but not in HPRD or HCPIN is difficult to evaluate due to the lack of gold standard.

### The database website

**Fig. 4:**
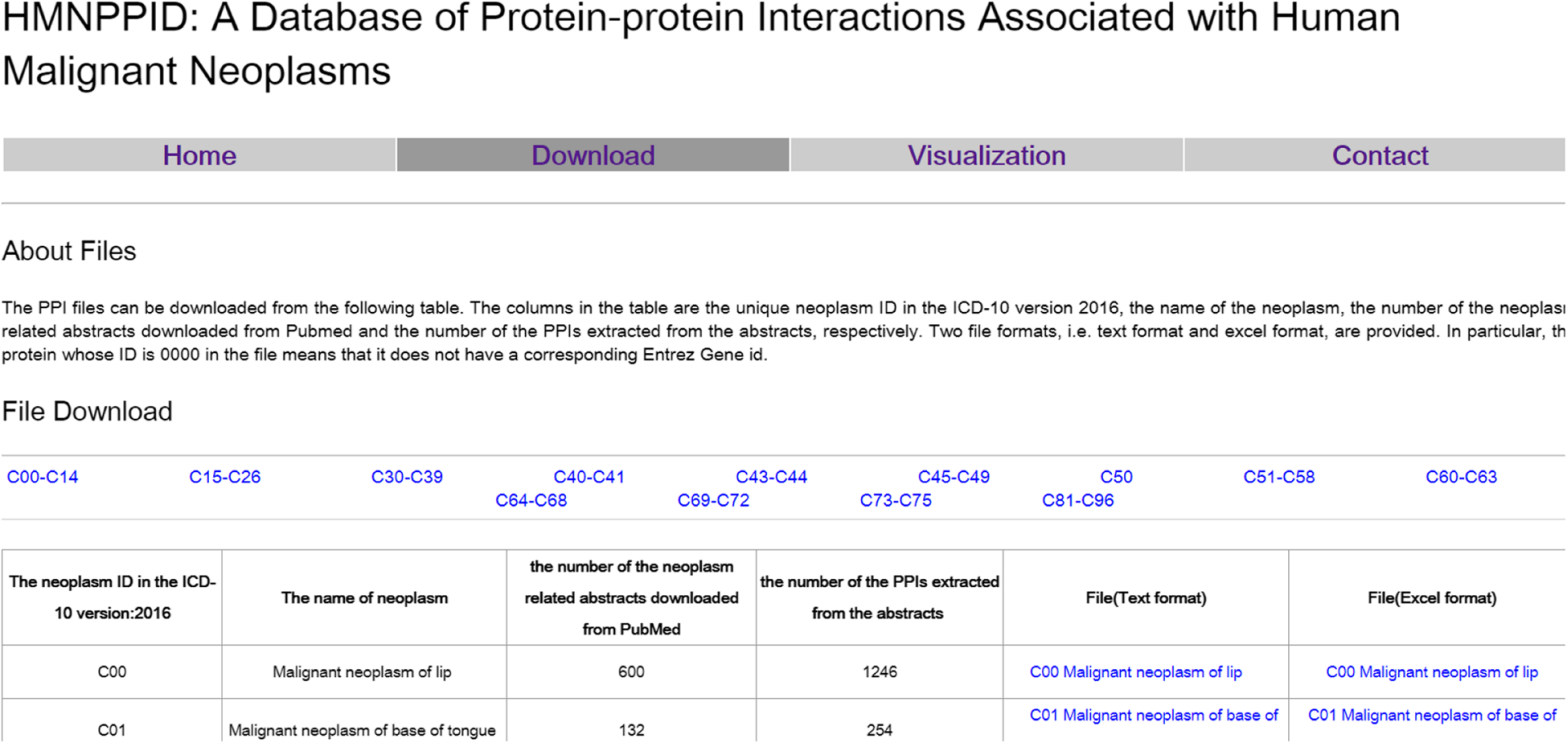
The web interface of HMNPPID database

As has been mentioned in the previous section, the PPIs of 171 types of malignant neoplasms were extracted with PPIExtractor, and then used to construct the PPI database of human malignant neoplasms, HMNPPID. HMNPPID can be accessed through http://202.118.75.18:8082/HMNPPID.asp. As shown in Fig. 4, on the web site the PPIs files are presented in tabular form. For each malignant neoplasm, the number of abstracts retrieved from PubMed with a corresponding query string and the number of the PPIs extracted from these abstracts is provided

**Fig. 5:**
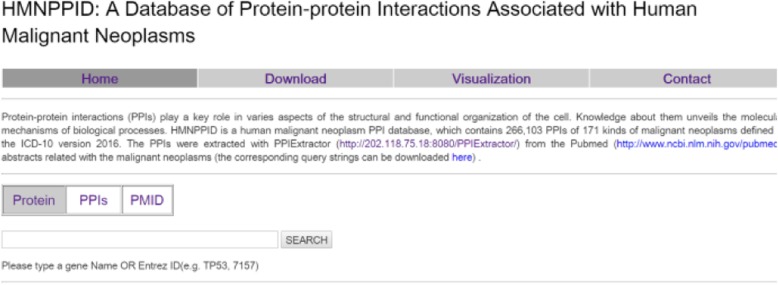
The query interface of HMNPPID database

In addition, the website also supports the query function (the query interface is shown in Fig. 5). Users can search the PPIs by the protein names (or Entrez IDs), protein name (or Entrez ID) pairs, and PubMed IDs.

### PPI visualization program

**Fig. 6:**
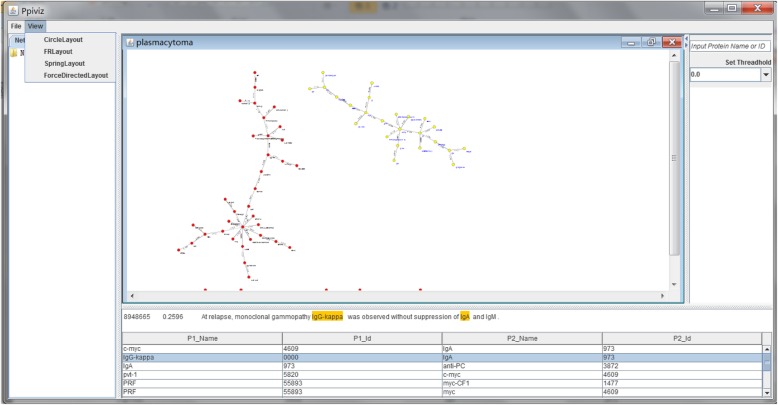
The interface of VisualPPI

To facilitate users to analyze the PPIs of specific malignant neoplasm, the PPI visual analysis tool is needed. Though there have been some existing visual approaches to PPI analysis such as STRING-DB [37], we provide a visualization tool of our own, called VisualPPI, because it is more convenient to display the detailed information about the PPI data in HMNPPID. It can be downloaded from the HMNPPID website (its interface is shown in Fig. 6). While opening a PPI file (text format) of a malignant neoplasm in VisualPPI, a PPI network is displayed. The nodes in the network represent the proteins and the edges represent that this pair of proteins interacting with each other.

**Fig. 7:**
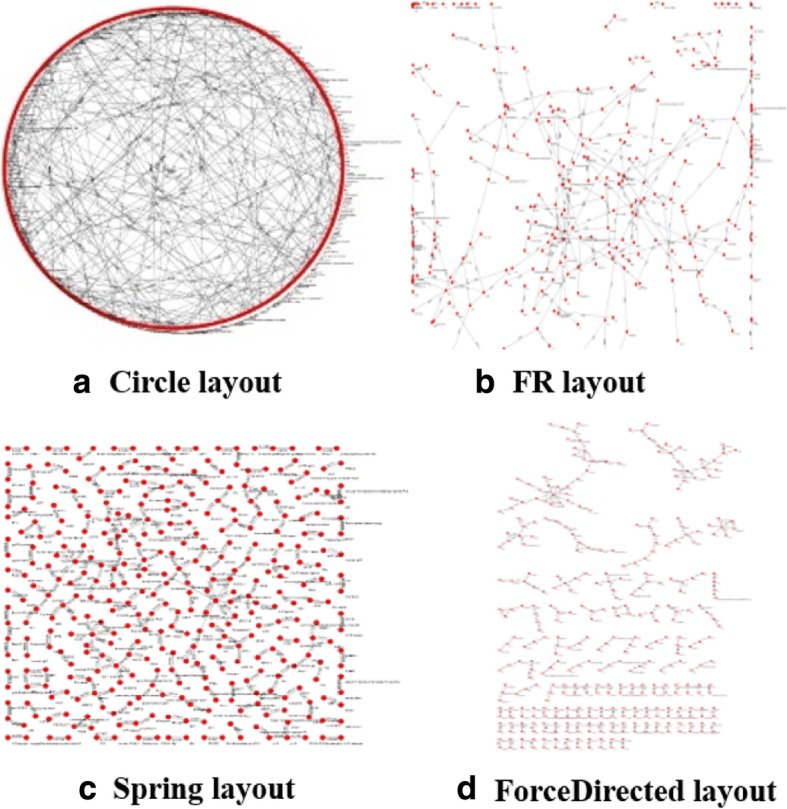
Four graphical display modes

VisualPPI provides four graphical display modes named “Circle layout,” “FR layout,” “Spring layout,” and “ForceDirected layout” (as shown in Fig. 7). In addition, the users can set the PPI filtering threshold as needed and the default value is 0, which indicates that only the PPIs whose confidence scores higher than 0 will be displayed in the network. For example, in Fig. 6, the display mode is “ForceDirected layout” and the threshold is set to 0. Selecting any region in the network (when the nodes change from red to yellow), users can get detailed information about PPIs at the bottom of the interface.

In our opinion, VisualPPI can facilitate the analysis of the specific PPI network of a malignant neoplasm and may help discover the molecular mechanisms behind the malignant neoplasm.

## Conclusions

The analysis on the PPIs of human malignant neoplasms helps unveil the molecular mechanisms behind. However, it is difficult to manually extract all the PPIs from large quantities of ever-growing biomedical literatures.

In this work, we constructed HMNPPID, a PPI database for human malignant neoplasms, using PPIExtractor from large amounts of biomedical texts. HMNPPID can hopefully become an important and readily available resource for the related research. We also provide the healthcare professionals with VisualPPI to help them efficiently analyze the PPI network of one specific malignant neoplasm.

As discussed in the “Background” section, currently, there have been some cancer-related PPI databases such as CancerNet and HCPIN. For CancerNet, it provides cancer-specific molecular interaction networks across multiple cancer types and the PPIs associated with a cancer are those of which the two pair mates were both found to be expressed in that cancer (genes were considered expressed if their transformed expression level was equal to or above 2 (in log2 (TPM + 1) scale) in at least 80% samples) [20]. By contrast, more types of human malignant neoplasm specific PPI data are provided in HMNPPID but are extracted from large quantities of PubMed abstracts with PPIExtractor.

For HCPIN, its interaction data are cancer-associated signaling pathways, but are not cancer-specific. In addition, they are a subset of the HPRD which was curated by expert biologists. Since the amount of biomedical literatures regarding PPIs is growing at an explosive speed, it is time-consuming and labor-intensive to manually extract PPIs from the unstructured texts. For HMNPPID, the PPIs associated with a cancer were extracted from the cancer-related PubMed abstracts with a tool PPIExtractor. On the one side, using PPIExtractor is much efficient than manual curation. For example, it only took about 8 days to extract 54,808 unique PPIs with the threshold 0 from 20,074 PubMed abstracts corresponding to the HPRD article set on a PC with an Intel i3-3220 CPU and 4G memory. On the other side, PPIExtractor can have satisfactory precision performance if a suitable threshold is set (usually the extracted PPI is reliable with the threshold 0). In fact, it achieved a precision of 79.23% on a DIP subset [27].

To keep the data up to date, we plan to update HMNPPID every half year (currently, the data in HMNPPID has been updated to April 30, 2019). In addition, our future research will focus on two areas in order to improve the quality and utility of the PPI database. First, we will improve the performance of PPIExtractor with the introduction of the popular deep learning method [38]. Second, we plan to extract the PPIs associated with human malignant neoplasms from full texts of the article instead of abstracts only which is recently made feasible with PMC Open Access BioC RESTful server (https://www.ncbi.nlm.nih.gov/research/bionlp/APIs/BioC-PMC/). As discussed in section *Evaluation of HMNPPID data*, this will improve the recall performance of PPI extraction. This paper is a revised and expanded version of a paper [39] presented at IEEE BIBM International Conference on Bioinformatics & Biomedicine (BIBM) 2018.

## Data Availability

HMNPPID is freely accessible at http://202.118.75.18:8082/HMNPPID.asp.

## Abbreviations

Bcl-2: B cell lymphoma 2
BIND: Biomolecular Interaction Network Database
BioGRID: Biological General Repository for Interaction Datasets
DIP: Database of Interacting Proteins
FABP4: Fatty acid-binding protein 4
FCG: Feature Coupling Generalization
HCPIN: Human Cancer Pathway Protein Interaction Network
HIPPIE: Human Integrated Protein–Protein Interaction rEference
HMNPPID: Human Malignant Neoplasm Protein–Protein Interaction Database
HPRD: Human Protein Reference Database
ICD: International Classification of Diseases
IID: Integrated Interaction Database
KEGG: Kyoto Encyclopedia of Genes and Genomes
MINT: Molecular INTeraction database
mTOR: Mammalian target of rapamycin
NER: Named Entity Recognition
PPIs: Protein–protein interactions
S6K1: Ribosomal S6 kinase 1
WHO: World Health Organization

## Footnotes

1 DIP: https://dip.doe-mbi.ucla.edu/

2 BIND: http://download.baderlab.org/BINDTranslation/

3 IntAct: https://www.ebi.ac.uk/intact/

4 HPRD: http://www.hprd.org/

5 BioGRID: https://thebiogrid.org/

6 HIPPIE: http://cbdm-01.zdv.uni-mainz.de/~mschaefer/hippie/

7 MINT: https://mint.bio.uniroma2.it/

8 MIPS: http://mips.gsf.de/proj/ppi/

9 IID: http://ophid.utoronto.ca/iid

10 STRING: http://string-db.org/

11 PathDIP: http://ophid.utoronto.ca/pathdip

12 CancerNet: http://bis.zju.edu.cn/CancerNet/

13 HCPIN: http://nesg.org:9090/HCPIN/

14 KEGG: https://www.genome.jp/kegg/

15 PubMed: https://www.ncbi.nlm.nih.gov/
